# Exploratory Research on Determinants of Place of Death in a Large-scale Cohort Study: The JPHC Study

**DOI:** 10.2188/jea.JE20210087

**Published:** 2023-03-05

**Authors:** Noriko Fujiwara, Naoki Shimada, Masanori Nojima, Keisuke Ariyoshi, Norie Sawada, Motoki Iwasaki, Shoichiro Tsugane

**Affiliations:** 1 Department of Palliative Medicine and Advanced Clinical Oncology, IMSUT Hospital, The Institute of Medical Science, The University of Tokyo, Tokyo, Japan; 2 Department of Biostatistics Graduate School of Medicine, Yokohama City University, Yokohama, Japan; 3 Department of Palliative Medicine, IMSUT Hospital, The Institute of Medical Science, The University of Tokyo, Tokyo, Japan; 4 Division of Advanced Medicine Promotion, The Institute of Medical Science, The University of Tokyo, Tokyo, Japan; 5 Japanese Organisation for Research and Treatment of Cancer, Tokyo, Japan; 6 Epidemiology and Prevention Group, Center for Public Health Sciences, National Cancer Center, Tokyo, Japan

**Keywords:** place of death, large-cohort study, factors influencing home death

## Abstract

**Background:**

The place of death and related factor, such as diseases, symptoms, family burden, and cost, has been examined, but social background and lifestyle were not considered in most studies. Here, we assessed factors that are associated with the place of death using the largest cohort study in Japan.

**Methods:**

A total of 17,781 deaths from the cohort study were assessed. The study database was created from the Japan Public Health Center-based Prospective Study (JPHC Study), in which demographic data were collected from Japanese Vital Statistics. Adjusted odds ratios for home death were calculated using logistic regression.

**Results:**

Multivariate analysis adjusted for various factors showed that unmarried status (odds ratio [OR] 2.4; 95% confidence interval [CI], 2.0–2.9), unemployed male (OR 1.3; 95% CI, 1.1–1.5), and high drinking level in male (OR 1.3; 95% CI, 1.1–1.6) were associated with home death. Regarding the cause of death, cardiovascular disease (OR 3.3; 95% CI, 2.9–3.8), cerebrovascular disease (OR 1.9; 95% CI, 1.6–2.2), and external factors (OR 4.1; 95% CI, 3.5–4.8) were significantly associated with home death, compared with cancer. The risk of death at home was significantly higher among unmarried subjects stratified by cause of death (cardiovascular disease: OR 3.2; 95% CI, 2.2–4.7; cerebrovascular disease: OR :5.1; 95% CI, 2.9–9.1; respiratory disease: OR 3.4; 95% CI, 1.6–7.6; and external factors: OR 2.3; 95% CI, 1.4–3.7), but for cancer, the risk of death at home tended to be higher among married participants.

**Conclusion:**

This study found that various factors are associated with home death using the largest cohort study in Japan. There is a high possibility of home deaths in people with fewer social connections and in those with diseases leading to sudden death.

## INTRODUCTION

Several recent reports have pointed out that there is a discrepancy between the desired place of death and the actual place of death in patients at the end of life.^1^^,^^2^ Numerous studies on the place of death have already been conducted, especially in cancer patients.^3^^,^^4^ For example, it was reported that cancer patients who died in hospitals and intensive care units had lower quality of life (QOL) compared to those who died in their homes in the United States.^5^ In addition, a recent systematic review reported that the gap between preferred place and actual place of death is larger in non-cancer patients than in cancer patients.^3^ On the other hand, although there have been several reports on the place of death based on large-scale demographic statistics, the number of studies analyzing the influence of social background and lifestyle is limited.^6^ In Japan, some studies regarding the place of death and its related factors have been conducted based on vital statistics from the Ministry of Health, Labour and Welfare.^2^^,^^7^ These related factors included family burden, cost, symptoms, and psychosocial issues; however, evaluation of individual lifestyle factors, such as marital status, social environment, economic background, and preferences, was not performed. Furthermore, no studies have assessed factors related to place of death based on large-scale cohort data in Japan.

Since the place of death has a great impact on the QOL at the end stages of life, clarifying factors associated with the place of death may contribute to improving the QOL of the entire population. By revealing the factors related to the place of death and its status, patients can understand the disincentives, risks, and important points for being at the desired place of death and prepare for it. Therefore, to address this, we assessed factors associated with the place of death in Japan, in order to provide optimal support for patients’ decision-making at the end of their lives using the data in the Japan Public Health Center-based Prospective Study (JPHC Study).

## METHODS

### Subjects

The JPHC Study consists of two cohorts: Cohort I was launched in 1990, and Cohort II was initiated in 1993. The details of the study were described in a previous report.^8^ The participants in cohort I are residents aged 40 to 59 years in five public health center areas, whereas participants in cohort II are residents aged 40 to 69 years living in six public health center areas. Confirmed dead cases were extracted from Cohort I and Cohort II. There were 17,781 deaths in Cohort I and Cohort II (male, 11,464; female, 6,317) between 1990 and 2011. Of these, 2,499 deaths (14.1%) occurred at home and care facilities, and 15,282 deaths (85.9%) occurred in a hospital.

### Death certification and data collection

We followed the death or migration of participants in the JPHC study through the residential registry in the municipality of each study area. Regarding subjects who migrated out of the study area, the coordinating center of the JPHC study asked the local government office of the participant’s new address about his/her vital status. Information on cause of death was obtained from death certificates with permission of the Ministry of Health, Labour and Welfare. Age at death, sex, marital status, whether the participant lived alone or not, cause of death, and death location were collected from the information in the death certificate. Other lifestyle factors, including occupation, smoking/drinking history, and physical activity, were obtained from the baseline questionnaire that had been administered when subjects were enrolled in the JPHC study.

The cause of death was coded using the International Statistical Classification of Diseases and Related Health Problems, 9^th^ revision (ICD-9), provided by World Health Organization, up through June 1995 and cause of death was coded using ICD-10 starting in July 1995. We classified the cause of death as “cancer” when coded as 140–239 in ICD-9 or C00–D48 in ICD-10, “cardiovascular” when coded as 390–459 in ICD-9 or I00–I99 in ICD-10, “respiratory” when coded as 460–519 in ICD-9 or J00–J99 in ICD-10, and “external” when coded as 800–999 in ICD-9 or S00–T98 in ICD-10. If coded as 430–438 in ICD-9 or I60–69 in ICD-10, “cardiovascular” was replaced by “cerebrovascular”, and “other” was assigned for all other causes of death.

As described above, this study was conducted as a case-control study using the information of the baseline and death of participants with confirmed deaths in the cohort study, in which those died at home were defined as cases and the others as controls.

### Ethical concerns

Participants of the JPHC study were informed of the objectives of the study and that completion of the survey questionnaire was regarded as providing consent to participate. However, since the subjects were already deceased at the beginning of this study, specific consent for this study was not obtained. This study was approved by the ethics committees of the National Cancer Center (Approval number: 2015-119) and the Institute of Medical Science, the University of Tokyo (Approval number: 26-89-0108).

### Statistics

Death at home (which included death at home, at a geriatric health services facility, or at a nursing home) was defined as the event, and odds ratios (ORs) of the event were calculated for surveyed items (age [continuous], sex, marital status [married unmarried, widowed, and divorced], living alone, occupation, smoking [never, current or ever], alcohol drinking [1–3/month, 1–2/week, 3–4/week, and ≥5/week], sports [1–3/month, 1–2/week, 3–4/week, and ≥5/week], cause of death [cancer, cardiovascular, cerebrovascular, respiratory, external, and other], and area). Fisher’s exact test (for 2 × 2 tables), chi-squared test (for multiple group comparisons) and Cochran-Armitage for trend (for ordinal variables) were used to test the significance of the difference and linear trend of the distribution of the outcome between categorized groups. Logistic regression analysis for the risk of the event was performed to calculate the OR adjusted for the factors listed above as potential confounders. We then performed a similar logistic regression analysis, in which the effect of marital status was assessed with stratification by the cause of death. *P* values for interaction were calculated by including the interaction terms between all the factors listed above and the cause of death into the model in which home death was included as dependent variable. The factors were included as categorical variables, and *P* values via type 3 Wald test were evaluated as *P* for interaction. All statistical analyses were performed using SAS 9.4 (SAS institute, Cary, NC, USA). *P* < 0.05 was considered statistically significant.

## RESULTS

Table 1 shows the sociodemographic characteristics of the deceased subjects in this study. Among males, younger men (<50 years) showed a relatively high proportion of home deaths, and there was an inverse relationship between the proportion of home deaths and 10-year age group among men under 80 years of age. However, in the age group of 80 years and over, the proportion of home deaths was relatively high in both men and women. Also, the proportion of home deaths was higher in unmarried individuals, divorced individuals, those living alone, and unemployed individuals, and this tendency was remarkable in males. With regard to lifestyle, there was no statistically significant difference in proportion of home deaths according to whether individuals did or did not have a smoking history, while the proportion of home deaths tended to be higher in the group with high drinking level (*P* = 0.068). With regard to sports and/or exercise, the higher the level of engaging in sports and/or exercise, the higher the proportion of home deaths (*P* = 0.037). Comparisons by the cause of death showed that deaths from cardiovascular disease, cerebrovascular disease, or external factors (eg, injury, addiction, and trauma) were more likely to occur at home. On the other hand, deaths from cancer and respiratory disease were less likely to occur at home.

**Table 1.** Sociodemographic characteristics of deceased persons by place of death

	Characteristics	Place of death; number (%) of people *N* = 17,781						Male *N* = 11,464						Female *N* = 6,317					
		
		Hospital	%	Home	%	Total	*P* value^*^	Hospital	%	Home	%	Total	*P* value^*^	Hospital	%	Home	%	Total	*P* value^*^
Total		15,282	(85.9)	2,499	(14.1)	17,781		9,862	(86.0)	1,602	(14.0)	11,464		5,420	(85.8)	897	(14.2)	6,317	

Age, years							0.238						0.007						0.138
	<50	333	(82.6)	70	(17.4)	403		206	(80.5)	50	(19.5)	256		127	(86.4)	20	(13.6)	147	
	50–59	2,127	(84.3)	396	(15.7)	2,523		1,400	(83.6)	275	(16.4)	1,675		727	(85.7)	121	(14.3)	848	
	60–69	4,774	(86.5)	744	(13.5)	5,518		3,232	(86.3)	512	(13.7)	3,744		1,542	(86.9)	232	(13.1)	1,774	
	70–79	5,959	(86.8)	903	(13.2)	6,862		3,853	(87.4)	554	(12.6)	4,407		2,106	(85.8)	349	(14.2)	2,455	
	≥80	2,089	(84.4)	386	(15.6)	2,475		1,171	(84.7)	211	(15.3)	1,382		918	(84.0)	175	(16.0)	1,093	

Sex							0.679						—						—
	Male	9,862	(86.0)	1,602	(14.0)	11,464		—		—				—		—			
	Female	5,420	(85.8)	897	(14.2)	6,317		—		—				—		—			

Marital status							<0.001						<0.001						<0.001
	Married	11,223	(88.0)	1,531	(12.0)	12,754		8,152	(88.2)	1,087	(11.8)	9,239		3,071	(87.4)	444	(12.6)	3,515	
	Unmarried	685	(72.5)	260	(27.5)	945		399	(69.9)	172	(30.1)	571		286	(76.5)	88	(23.5)	374	
	Widowed	2,718	(84.0)	516	(16.0)	3,234		910	(81.3)	209	(18.7)	1,119		1,808	(85.5)	307	(14.5)	2,115	
	Divorced	645	(77.2)	191	(22.9)	836		393	(74.6)	134	(25.4)	527		252	(81.6)	57	(18.4)	309	

Living alone							<0.001						<0.001						<0.001
	Yes	842	(78.3)	234	(21.8)	1,076		388	(75.3)	127	(24.7)	515		454	(80.9)	107	(19.1)	561	
	No	14,242	(86.5)	2,229	(13.5)	16,471		9,348	(86.5)	1,458	(13.5)	10,806		4,894	(86.4)	771	(13.6)	5,665	

Occupation							0.009						0.002						0.438
	Yes	11,422	(86.4)	1,797	(13.6)	13,219		8,384	(86.5)	1,313	(13.5)	9,697		3,038	(86.3)	484	(13.7)	3,522	
	No	3,568	(84.8)	640	(15.2)	4,208		1,281	(83.5)	254	(16.5)	1,535		2,287	(85.6)	386	(14.4)	2,673	

Smoking							0.786						0.679						0.582
	Never	8,027	(86.0)	1,306	(14.0)	9,327		3,241	(86.2)	518	(13.8)	3,759		4,786	(85.9)	788	(14.1)	5,574	
	Current or Ever	7,125	(85.9)	1,173	(14.1)	8,293		6,549	(85.9)	1,072	(14.1)	7,621		576	(85.1)	101	(14.9)	677	

Alcohol drinking							0.068						0.026						0.263
	Non-drinkers	6,704	(86.5)	1,045	(13.5)	7,749		2,300	(87.4)	333	(12.6)	2,633		4,404	(86.1)	712	(13.9)	5,116	
	1–3/month	1,058	(86.4)	166	(13.6)	1,224		701	(87.1)	104	(12.9)	805		357	(85.2)	62	(14.8)	419	
	1–2/week	819	(84.6)	149	(15.4)	968		636	(84.2)	119	(15.8)	755		183	(85.9)	30	(14.1)	213	
	3–4/week	1,094	(86.2)	175	(13.8)	1,269		939	(86.6)	145	(13.4)	1,084		155	(83.8)	30	(16.2)	185	
	≥5/week	5,131	(85.4)	875	(14.6)	6,006		4,888	(85.5)	830	(14.5)	5,718		243	(84.4)	45	(15.6)	288	

Sports and/or physical exercise							0.037						0.132						0.141
	No exercise	10,967	(86.2)	1,758	(13.8)	12,725		6,889	(86.1)	1,111	(13.9)	8,000		4,078	(86.3)	647	(13.7)	4,725	
	1–3 days/month	1,355	(87.0)	202	(13.0)	1,557		1,082	(87.5)	154	(12.5)	1,236		273	(85.0)	48	(15.0)	321	
	1–2 days/week	1,087	(85.2)	189	(14.8)	1,276		703	(85.6)	118	(14.4)	821		384	(84.4)	71	(15.6)	455	
	3–4 days/week	629	(83.6)	123	(16.4)	752		410	(85.8)	68	(14.2)	478		219	(79.9)	55	(20.1)	274	
	≥5 days/week	977	(84.7)	176	(15.3)	1,153		621	(83.6)	122	(16.4)	743		356	(86.8)	54	(13.2)	410	

Cause of death							<0.001						<0.001						<0.001
	Cancer	6,910	(91.9)	610	(8.1)	7,520		4,477	(91.8)	400	(8.2)	4,877		2,433	(92.1)	210	(7.9)	2,643	
	Cardiovascular	2,065	(75.3)	679	(24.7)	2,744		1,308	(75.3)	430	(24.7)	1,738		757	(75.3)	249	(24.8)	1,006	
	Cerebrovascular	1,563	(83.8)	302	(16.2)	1,865		942	(82.6)	198	(17.4)	1,140		621	(85.7)	104	(14.3)	725	
	Respiratory	1,616	(92.6)	129	(7.4)	1,745		1,144	(93.3)	82	(6.7)	1,226		472	(90.9)	47	(9.1)	519	
	External	1,087	(70.8)	448	(29.2)	1,535		797	(73.7)	285	(26.3)	1,082		290	(64.0)	163	(36.0)	453	
	Other	2,041	(86.1)	331	(14.0)	2,372		1,194	(85.2)	207	(14.8)	1,401		847	(87.2)	124	(12.8)	971	

Death location (percentage in *N*)																			
	
“Home death” including	Home			2,280	(12.8)					1,485	(13.0)					795	(12.6)		
	Geriatric Health Services Facility			57	(0.3)					35	(0.3)					22	(0.4)		
	Nursing home			162	(0.9)					82	(0.7)					80	(1.3)		
“Hospital or other death” including	Hospital	14,114	(79.4)					9,068	(79.1)					5,046	(79.9)				
	Clinic	646	(3.6)					371	(3.2)					275	(4.4)				
	Other	522	(2.9)					423	(3.7)					99	(1.6)				

^*^Fisher’s exact test (for 2 × 2 tables), chi-squared test (for multiple group comparisons) and Cochran-Armitage for trend (for ordinal variables) were used to test the significance of the difference.

Table 2 shows the results of multivariate analysis for home death (ie, death at home, at a geriatric health services facility, or at a nursing home). As a result, there were no associations between age or sex and place of death. As for marital status, there was a statistically significant association between unmarried state and death at home in the study population (OR 2.40; 95% CI, 1.98–2.92). There were significant associations between being widowed or divorced and death at home only in males (OR 1.75; 95% CI, 1.45–2.13 and OR 2.35; 95% CI, 1.82–3.04, respectively). There was no significant association between living alone and death at home. Regarding occupation, there was a significant association between unemployed state and death at home in males (OR 1.28; 95% CI, 1.07–1.53). In addition, there was a significant association between drinking alcohol more than five times a week and home death in males (OR 1.32; 95% CI, 1.13–1.55). Regarding the cause of death, cardiovascular disease (OR 3.30; 95% CI, 2.88–3.77), cerebrovascular disease (OR 1.86; 95% CI, 1.57–2.20), and external factors (eg, injury, addiction, and trauma) (OR 4.13; 95% CI, 3.52–4.84) were more strongly associated with death at home than cancer.

**Table 2.** Logistic regression analysis of liklihood of dying in a home

Variables	Total		Male		Female		Home death (excluding geriatric health services facility/nursing home)	
			
	OR	95% CI	OR	95% CI	OR	95% CI	OR	95% CI
Age at death, years								
<50	1		1		1		1	
50–59	1.03	0.71–1.51	1.02	0.64–1.62	1.04	0.53–2.03	1.02	0.70–1.49
60–69	1.00	0.69–1.44	0.98	0.62–1.54	1.00	0.53–1.91	0.97	0.67–1.41
70–79	0.97	0.67–1.40	0.95	0.60–1.50	1.00	0.53–1.90	0.87	0.60–1.26
≥80	1.11	0.76–1.63	1.11	0.69–1.80	1.14	0.59–2.20	0.82	0.56–1.21

Sex								
Male	1						1.00	
Female	1.03	0.89–1.18					1.02	0.88–1.18

Marital status								
Married	1.00		1.00		1.00		1.00	
Unmarried	2.40	1.98–2.92	2.78	2.17–3.57	1.81	1.32–2.50	2.21	1.81–2.70
Widowed	1.40	1.22–1.60	1.75	1.45–2.13	1.09	0.90–1.32	1.33	1.15–1.53
Divorced	1.92	1.56–2.37	2.35	1.82–3.04	1.26	0.86–1.85	1.89	1.53–2.34

Living alone								
Yes	1.00		1.00		1.00		1.00	
No	0.91	0.75–1.10	0.96	0.72–1.27	0.82	0.62–1.09	0.90	0.73–1.11

Occupation								
Yes	1.00		1.00		1.00		1.00	
No	1.13	1.00–1.27	1.28	1.07–1.53	1.01	0.85–1.19	1.14	1.01–1.30

Smoking								
Never	1.00		1.00		1.00		1.00	
Current or ever	0.95	0.85–1.07	0.99	0.87–1.14	0.85	0.65–1.12	1.00	0.88–1.13

Alcohol drinking								
Non-drinkers	1.00		1.00		1.00		1.00	
1–3/month	1.05	0.86–1.29	0.99	0.76–1.30	1.17	0.85–1.62	1.05	0.85–1.30
1–2/week	1.15	0.92–1.44	1.28	0.98–1.67	0.79	0.48–1.29	1.19	0.95–1.50
3–4/week	1.11	0.90–1.36	1.11	0.87–1.41	1.34	0.86–2.09	1.14	0.92–1.41
≥5/week	1.28	1.11–1.47	1.32	1.13–1.55	1.23	0.84–1.80	1.26	1.09–1.45

Sports and/or physical Exercise								
Rarely	1.00		1.00		1.00		1.00	
1–3 days/month	0.97	0.81–1.15	0.91	0.74–1.11	1.24	0.88–1.76	1.01	0.85–1.21
1–2 days/week	1.16	0.96–1.39	1.09	0.87–1.38	1.28	0.95–1.72	1.14	0.94–1.38
3–4 days/week	1.25	1.00–1.56	1.03	0.76–1.39	1.63	1.16–2.28	1.16	0.92–1.47
≥5 days/week	1.15	0.95–1.39	1.28	1.02–1.61	0.93	0.67–1.28	1.17	0.96–1.42

Cause of death (the same as model 2)								
Cancer	1.00		1.00		1.00		1.00	
Cardiovascular	3.30	2.88–3.77	3.13	2.65–3.71	3.70	2.96–4.63	3.16	2.76–3.62
Cerebrovascular	1.86	1.57–2.20	1.88	1.51–2.33	1.87	1.41–2.47	1.67	1.40–1.99
Respiratory	0.78	0.63–0.97	0.66	0.50–0.87	1.05	0.73–1.51	0.56	0.43–0.73
External	4.13	3.52–4.84	3.51	2.88–4.28	5.90	4.50–7.75	4.10	3.50–4.82
Other	1.61	1.37–1.89	1.61	1.31–1.97	1.65	1.27–2.13	1.36	1.15–1.61

CI, confidence interval; OR, odds ratio.

Adjusted for age, sex, marital status, living alone, occupation, smoking, alcohol drinking, sports, cause of death, and area.

Next, to assess the heterogeneity of the effect of factors related to home death indicated across the causes of death, we thoroughly evaluated *P* values for the interactions between the factors and the causes followed by stratified analyses (Table 3). As a result, significant interactions of marital status and age with cause of death (*P* < 0.001 for both interaction) were observed. Except in those with cancer, the risk of death at home was significantly higher among unmarried participants, even with adjustment for various factors (cardiovascular disease: OR 3.19; 95% CI, 2.17–4.68; cerebrovascular disease: OR 5.10; 95% CI, 2.86–9.10; respiratory disease: OR 3.43; 95% CI, 1.55–7.61; and external factors: OR 2.28; 95% CI, 1.40–3.70). In those with cardiovascular disease, cerebrovascular disease, or external factors as the cause of death, significant associations were also found between widowed state (cardiovascular disease: OR 1.76; 95% CI, 1.35–2.29; cerebrovascular disease: OR 2.07; 95% CI, 1.40–3.06; external factors: OR 1.75; 95% CI, 1.19–2.58) or divorced state (cardiovascular disease: OR 2.76; 95% CI, 1.81–4.20; cerebrovascular disease: OR 3.07; 95% CI, 1.67–5.66; external factors: OR 1.71; 95% CI, 1.01–2.89) and death at home. In contrast, among those with cancer, the influence of marital status was less than that in other causes of death, and married status rather tended to be associated with home death (ORs 0.63–0.76 in non-married status). In addition, when we combined non-married statuses, the direction of the effect in the married population was clearly opposite in those with cancer (OR 0.74; 95% CI, 0.57–0.96) compared to the other causes. In addition, ORs in the elderly age group compared to the youngest group indicated a positive association between age and home death in those with cancer (OR per 10-year increase in age, 1.23; 95% CI, 1.10–1.37), but not in the other populations.

**Table 3.** Odds of home death according to marital status by cause of death

Cause of death	Married	Unmarried				Widowed				Divorced				Non-married^a^				*P* for interaction^b^
				
	(Reference)		95% CI				95% CI				95% CI				95% CI			
				
	OR	OR	Lower		Upper	OR	Lower		Upper	OR	Lower		Upper	OR	Lower		Upper	
Cancer	1	0.75	0.43	–	1.31	0.76	0.57	–	1.01	0.63	0.35	–	1.12	0.74	0.57	–	0.96	*P* < 0.001
Cardiovascular	1	3.19	2.17	–	4.69	1.76	1.35	–	2.29	2.76	1.81	–	4.20	2.17	1.74	–	2.71	
Cerebrovascular	1	5.10	2.86	–	9.10	2.07	1.40	–	3.06	3.07	1.67	–	5.66	2.64	1.9	–	3.66	
Respiratory	1	3.43	1.55	–	7.61	1.42	0.85	–	2.37	1.35	0.48	–	3.77	1.73	1.11	–	2.71	
External	1	2.28	1.40	–	3.70	1.75	1.19	–	2.58	1.71	1.01	–	2.89	1.82	1.35	–	2.44	
Other	1	2.99	1.85	–	4.81	1.58	1.12	–	2.23	4.07	2.48	–	6.67	2.23	1.67	–	2.98	

Cause of death	Age <50	Age 50–59				Age 60–69				Age 70–79				Age ≥80				Age per 10-year increase				*P* for interaction^c^
					
	(Reference)		95% CI				95% CI				95% CI				95% CI				95% CI			
					
	OR	OR	Lower		Upper	OR	Lower		Upper	OR	Lower		Upper	OR	Lower		Upper	OR	Lower		Upper	
Cancer	1	1.23	0.43	–	3.54	1.45	0.52	–	4.04	1.80	0.64	–	5.01	2.27	0.80	–	6.49	1.23	1.10	–	1.37	<0.001
Cardiovascular	1	0.82	0.35	–	1.89	0.73	0.33	–	1.64	0.61	0.27	–	1.35	0.66	0.29	–	1.49	0.91	0.81	–	1.01	
Cerebrovascular	1	2.06	0.44	–	9.71	1.86	0.41	–	8.46	1.74	0.39	–	7.86	1.47	0.32	–	6.76	0.93	0.80	–	1.09	
Respiratory	1	0.14	0.02	–	0.87	0.17	0.04	–	0.79	0.22	0.05	–	0.94	0.24	0.06	–	1.07	1.05	0.82	–	1.35	
External	1	1.03	0.53	–	2.00	1.00	0.52	–	1.90	0.65	0.33	–	1.26	0.35	0.15	–	0.80	0.77	0.67	–	0.89	
Other	1	0.88	0.33	–	2.35	0.70	0.27	–	1.82	0.51	0.20	–	1.32	0.87	0.33	–	2.30	0.96	0.84	–	1.11	

CI, confidence interval; OR, odds ratio.

OR was adjusted by age, sex, marital status, living alone, occupation, smoking, alcohol drinking, sports, cause of death, and area.

^a^“Non-married” was defined as the combination of unmarried, widowed and divorced (marital status was categorized into two categories for the analysis comparing married vs non-married).

^b^Interaction between cause of death and married status (with four categories: married, unmarried, widowed, and divorced) for home death.

^c^Interaction between cause of death and age (as categorical variable with five age categories) for home death.

## DISCUSSION

This is the first study to clarify the place of death and related factors, including information on lifestyle (such as various social backgrounds and preferences) using a large-scale cohort study in Japan. Overall, the results implied an association between home death and less social involvement due to marital status and occupation, and associations between home death and particular diseases in which it is more difficult to predict the timing of death. Regarding lifestyle factors, there were significantly higher risks for home death due to frequent drinking and exercise habits, but smoking was not related to home death.

### Marital status

Our results showed that unmarried, widowed, and divorced states were strongly related to home death, especially in unmarried males. A previous study showed that married people were more likely to die at home in the results of the study using death certificate data from 14 countries in cancer patients.^9^ On the other hand, studies in Canada and Portugal have reported that a non-married state was associated with home death.^10^^,^^11^

Marital status is one of the most influential factors in social life. However, no previous studies examined the relationship between marital status and place of death analyzed by the cause of death. In our study, a strong association between a non-married state and home death was observed among those whose cause of death was cardiovascular or cerebrovascular disease. These diseases often lead to sudden death, unlike cancer,^12^ especially without bystander support. In such cases, the patient will not be transported to the hospital and will die at home. Our findings suggest the usefulness of further research investigating what services (medical or social) are needed for people with each disease. In contrast, our results indicated that married status rather tended to be associated with home death in cancer patients, and this can be facilitated by the presence of home caregivers who provide care for patients at home.

### Cause of death

As noted above, the relationship between marital status and place of death varied according to the cause of death. In cancer patients, we showed that there was no significant association between marital status and home death. According to our results, the proportion of home deaths was low in cancer patients, and it did not increase even with non-married status. In contrast, the proportion of home deaths was high among patients with all diseases except cancer in the non-married state compared with the married state in our study. One of the reasons for this result could be due to a high possibility that cancer patients are less likely to die even if they are transported by emergency medical services because their physical function gradually declines or the declines are predictable in non-acute diseases, such as cancer. In Japan, approximately 60% of patients who are emergently transported are elderly patients, and patients who were dead on arrival comprised 1.3%, which means that the number of surviving patients who were emergently transported was high, and 50% of the emergently transported patients required hospitalization.^13^ In addition, Japan had the highest rate of hospital beds per 1,000 population and the second longest hospital stays among countries in the Organisation for Economic Co-operation and Development (OECD) (“OECD Health Statistics 2019 - OECD,” n.d.).^14^ Considering the above, most cancer patients are taken care of in a hospice or general ward after emergency transportation at the end of their lives. However, according to the result shown in Table 3, married and elderly cancer patients tended to die at home, although the proportion of home death was lower in cancer than in some of the other causes, such as cardiovascular diseases. It can be interpreted that married or elderly cancer patients who have family support and do not seek aggressive treatment may choose to receive end-of-life care at home.

In Japan, with an aging society, there are increasing numbers of socially isolated people and people who suffer solitary deaths. According to surveys in Tokyo, of the 78,278 deaths in 2018, 13,118 (16.8%) were “unnatural deaths” (many of these deaths had no bystanders when they died) and about 6,444 (half of unnatural deaths) were living alone. Furthermore, most unnatural deaths were caused by cardiovascular disease.^15^ In our findings, out of 2,499 home deaths, the largest number (679) died at home due to cardiovascular disease. These findings are consistent with previous reports.^16^^,^^17^ Although our study did not examine whether there was a gap between preferred and actual place of death or not, it might be necessary to carefully consider whether it means desired place of living until the time of death, or desired place of death (the moment of death), especially in Japan.

### Preference and work/lifestyle

In the findings related to preference and lifestyle, while there was no significant difference in the proportion of home deaths according to the presence or absence of a smoking history, a positive association between home death and drinking was observed. There might be some factors related to delays in finding incidents or calling emergency transportation, because drinking and home deaths were independently associated even after adjustment for multiple factors. The proportion of home deaths was also high in the group who engage in frequent sports and/or physical exercise, but it is not clear whether this is a true association or a result based on the lack of adjustment for confounders.

Some reports have shown that the risk factors for solitary death were “male, unemployed, and living alone”.^17^^,^^18^ Our findings also showed that unmarried and unemployed men had a high possibility of home deaths, similar to previous studies. Since there are decreasing social connections in an aged society, great attention should be paid to solitary death.

### Limitations

The cause of death, gender, age, and marital status were at the time of their deaths. However, since the lifestyle characteristics were obtained only at the start of the cohort survey, we could not study the lifestyle characteristics of the subjects just before the time of their death. Nonetheless, our study had remarkable advantages as follows: it was based on a large-scale cohort study; with lifestyle characteristics of the subjects, including their marital status, social environment, economic background, and preferences; and the cause of death was accurate and obtained from their death certificate. This study identified the place of death and associated factors. The place where people spend their final days could be considered in future studies.

### Implementation of practice

In summary, this study showed that there was a high possibility of home death in people with fewer social connections, such as non-married status or unemployed status, particularly in men. The possibility of home death was extremely high in those with cardiovascular disease, and this result is similar to previous reports. Among cancer patients, there were few deaths at home, suggesting that a high percentage of cancer patients received medical services at a hospital at the end of life.

In an aging and aged society, there is a growing demand for social support for people with few connections with society, such as those who live alone, and there is a risk of sudden death in those with cardiovascular disease.
